# Diversity, Bacterial Symbionts and Antibacterial Potential of Gut-Associated Fungi Isolated from the *Pantala flavescens* Larvae in China

**DOI:** 10.1371/journal.pone.0134542

**Published:** 2015-07-29

**Authors:** Ming-Wei Shao, Yi-Hui Lu, Shuang Miao, Yun Zhang, Ting-Ting Chen, Ying-Lao Zhang

**Affiliations:** 1 College of Chemistry and Life Science, Zhejiang Normal University, Jinhua, 321004, PR China; 2 State Key Laboratory of Pharmaceutical Biotechnology, Nanjing University, Nanjing, 210093, People’s Republic of China; University of Camerino, ITALY

## Abstract

The diversity of fungi associated with the gut of *Pantala flavescens* larvae was investigated using a culture-dependent method and molecular identification based on an analysis of the internally transcribed spacer sequence. In total, 48 fungal isolates were obtained from *P*. *flavescens* larvae. Based on phylogenetic analyses, the fungal isolates were grouped in 5 classes and 12 different genera. Fourteen bacterial 16S rDNA sequences derived from total genomic DNA extractions of fungal mycelia were obtained. The majority of the sequences were associated with Proteobacteria (13/14), and one Bacillaceae (1/14) was included. *Leclercia* sp., *Oceanobacillus oncorhynchi* and *Methylobacterium extorquens*, were reported for the first time as bacterial endosymbionts in fungi. High-performance liquid chromatography (HPLC) analysis indicated that bacterial symbionts produced specific metabolites and also exerted an inhibitory effect on fungal metabolites. The biological activity of the fungal culture extracts against the pathogenic bacteria *Staphylococcus aureus* (ATCC 6538), *Bacillus subtilis* (ATCC 6633) and *Escherichia coli* (ATCC 8739) was investigated, and 20 extracts (42%) exhibited antibacterial activity against at least one of the tested bacterial strains. This study is the first report on the diversity and antibacterial activity of symbiotic fungi residing in the gut of *P*. *flavescens* larvae, and the results show that these fungi are highly diverse and could be exploited as a potential source of bioactive compounds.

## Introduction

Insects constitute the largest group of fauna on Earth and are found worldwide. Currently, approximately 1 million types of insects are known to science, but many unknown species await discovery. Insects from many different taxonomic groups harbor maternally transmitted microbial symbionts [1–4]. The enormous diversity of insects nurtures a large microbial community in the insect gut. Reports indicate that some microbial species may exert beneficial effects on the digestion and immune systems of insects, such as aphids [5], beewolf wasps [6], fungus-growing beetles [7] and fungus-growing ants [8], which are believed to benefit from gut-associated microorganisms that provide nutrition and defensive chemicals or modulate immune responses. However, in contrast to our understanding of gut-associated bacteria, which have been widely documented [9–13], little is known about the relationship between insects and their associated fungi. In addition, more and more studies are focused on the potential of bacterial-fungal symbioses with the aim of expounding the ecological role of fungi [14, 15]. For example, endobacteria, rather than the fungus itself, were reported to produce mycotoxins in *Rhizopus microsporus* [16, 17]. Therefore, the biological and evolutionary aspects of symbiotic relationships between fungi and insects, as well as between fungi and bacteria, should be studied without delay.

Direct evidence indicates that insect gut fungi will become an important source of active natural products and new microorganism resources. For example, the fungus *Curvularia* sp. FH01 was found to be an efficient producer of phytotoxic and antifungal compounds [18]. *Daldinia eschscholzii*, isolated from the gut of the mantis species *Tenodera aridifolia*, was reported to simultaneously generate four novel skeletons and seven structurally unique metabolites that have the potential to become immune inhibitors [19, 20]. In addition to its chemical diversity and as an important source of pharmacologically active compounds, the insect gut was also reported to be an important source of new microbial species. White et al. [21] described many new gut fungi in the course of seeking specimens of Harpellales from aquatic insect larvae in Norway. However, comprehensive, scientific and ecologically relevant investigations of the insect gut (especially in carnivorous and aquatic insects) are limited, especially when considering the large diversity of insect species.

*Pantala flavescens* is a carnivorous insect, and the larvae are fiercely carnivorous in aquatic environments [22]. In China, larvae of *P*. *flavescens* were customarily eaten as early as 3,000 years ago and are also used in traditional Chinese medicine. The larvae have a special mouthpart structure resembling a mask. When attacking prey, the larvae must rapidly fold the lower lip mask and front double saw to clamp onto the prey and then transfer the food into its mouth. During the feeding process, water containing bacteria and fungi are part of the natural diet of *P*. *flavescens* larvae. These microbes, which survive in the gut, can help provide some nutrition and chemical protection for the larvae. These surviving microbes, especially those from the filamentous fungi, can be exploited for drug discovery. However, our knowledge about the diversity and antibacterial potential of these surviving fungi is still limited, with many aspects awaiting study. Here, we report detailed information about the diversity, bacterial symbionts and antibacterial potential of gut-associated fungi isolated from the *Pantala flavescens* larvae.

## Materials and Methods

### Ethics Statement

In our study, only *P*. *flavescens* larvae were collected, and no specific permits were required for the field studies described. *P*. *flavescens* is widely distributed in Zhejiang and throughout China. They are the most common dragonflies in the world, so our work did not involve endangered or protected species.

### Sampling Sites

*P*. *flavescens* fifth-instar larvae were collected from a river near Zhejiang Normal University (29°00'17.37"N, 119°29'54.84"E) in Jinhua city of Zhejiang Province, PR China, in August 2012.

### Isolations of Symbiotic Fungi

Surface sterilization and isolation of symbiotic fungi were accomplished by following our previously established procedures [23]. Briefly, the samples were promptly transported to the laboratory and starved for 24 h. Prior to dissection, the larvae of *P*. *flavescens* were surface-sterilized by dipping them in 75% EtOH for 2 min, followed by rinsing in sterilized water. After dissection, the guts obtained from individual larvae (n = 10) were carefully homogenized with sterilized water. Then, the homogenates were diluted in a 10-fold series (i.e., 10^−1^, 10^−2^, 10^−3^), and aliquots of 200 μL from each dilution were spread onto plates containing malt-extract agar (MEA) (consisting of 20 g of malt extract, 20 g of sucrose, 1 g of peptone, 20 g of agar in 1 L of distilled water). Fungi were incubated aerobically in a chamber for 3 d at 28 ± 0.5°C, and pure colonies of fungi from the appropriate dilution were transferred into new MEA medium. Isolated strains were preserved on MEA slants at 4°C until use. Fungal species were grouped via molecular sequence data. The fungi were stored at our institute.

### DNA Sequencing

Before DNA extraction, fungal isolates were first cultured on MEA medium. Then, the fresh mycelia was transferred to a 250 mL Erlenmeyer flask containing 100 mL of ME medium (20 g of malt extract, 20 g of sucrose, 1 g of peptone, 1 L of distilled H_2_O) and cultured at 28°C on rotary shakers at 180 rpm for 3 d. The growing mycelia were used to provide samples for total DNA extraction. Fungal genomic DNA was extracted using the Fast DNA Extraction Kit for Plants (BioTeke, Beijing, China) according to the manufacturer’s instructions. The extracted DNA was stored at 4°C until further use.

The internally transcribed spacer regions were amplified using the primers ITS1 (5′-TCCGTAGGTGAACCTGCGG-3′) and ITS4 (5′-TCCTCCGCTTA TTGATATGC-3′) [24]. Amplification was accomplished in a 50 μL reaction mixture containing 1 μL of genomic DNA (approximately 50 ng), 0.2 μL of TaKaRa Taq (1 U) (Takara Biotechnology Co., Ltd., Dalian, China), 4 μL of reaction buffer (10×) and 4 μL of dNTPs (2.5 mM each) and 1 μL of each primer (10 μM), and 39 μL of sterile Milli-Q water. The PCR conditions were an initial denaturation step at 94°C for 2 min, followed by 35 cycles of 60 s of denaturation at 94°C, 60 s of primer annealing at 55°C, and 60 s of extension at 72°C, and 10 min at 72°C for final chain elongation. The size and purity of the PCR products were evaluated by 1% agarose gel electrophoresis. Qualified PCR products were selected for sequencing (Sangon Biotech Co., Ltd., Shanghai, China).

Samples of 16S rDNA were amplified using the 27F primers (5'-AGAGTTTGATCCTGGCTCAG-3') 1492R and primers (5'-GGTTACCTTGTTA-CGACTT-3') [25] based on 48 fungal genomic DNA. Amplification was accomplished in a 50 μL reaction mixture containing 1 μL of genomic DNA (approximately 50 ng), 0.5 μL of TaKaRa Taq (1 U) (Takara Biotechnology Co.,Ltd., Dalian, China), 5 μL of reaction buffer (10×) and 5 μL of dNTPs and 1 μL of each primer, and 34.5 μL of sterile Milli-Q water. The PCR conditions were 30 cycles at 94°C for 2 min, 55°C for 1 min, and 72°C for 2 min and 10 min at 72°C for a final chain elongation. PCR products of the expected size (approximately 1.5 kb) were evaluated on a 1% agarose gel electrophoresis. Qualified PCR products were selected for sequencing (Sangon Biotech Co., Ltd., Shanghai, China).

### Identification of Isolates and Phylogenetic Analyses

Preliminary identifications of all resulting sequences were compared with the available data in GenBank (http://blast.ncbi.nlm.nih.gov) using BLAST to determine their phylogenetic affiliation. Neighbor-joining analyses were performed by MEGA v5.0 after multiple alignments of the data using CLUSTAL-X, with gaps treated as missing data. Bootstrap analysis was used to evaluate the tree topology of the neighbor-joining data by performing 1,000 replicates [26]. The internally transcribed spacer sequence and bacterial 16S rDNA sequences reported in this paper have been submitted to GenBank.

### Confocal Laser Scanning Microscopy

According to the methods detailed by Partida-Martinez and Sulemankhil [16, 27], fresh fugal mycelia (0.5 mL) of QTYC-45 and symbiont-free QTYC45 were transferred to a centrifuge tube containing 0.5 mL of 0.85% saline. An aliquot of mycelia (10 μL) was placed onto a microscope slide, and then, 0.5 μL of Live/Dead BacLight SYTO 9 and an equal amount of propidium iodide stains (Live/Dead BacLight Bacterial Viability Kit, Molecular Probes-L7007) were used to stain the bacteria in the mycelia. After 15 min of incubation in the dark (RT), ProLong Gold antifade reagent (Molecular Probes—P36935) was used to enhance the fluorescence. The sample was incubated for another 20 min and analyzed using a Leica TCS SP5 AOBS Laser Microscope (Leica Microsystems Ltd., Wetzlar, Germany) at 480/500 nm.

### HPLC Analysis of Metabolite Profile of Fungi and Sterile Fungi

Ten pieces (5 mm in diameter) of QTYC45 and symbiont-free QTYC45 were inoculated into a 500 mL Erlenmeyer flask containing 150 mL of ME medium followed by culturing for 4 d at 180 rpm and 28°C. The culture broth filtrate (300 mL) was extracted with ethyl acetate (3 × 300 mL), and the mycelia were extracted overnight with methanol. All extracts were re-dissolved at a concentration of 2 mg/mL and monitored at 254 nm. High-performance liquid chromatography (HPLC) analyses were carried out on an Agilent 1260 HPLC (Agilent Technologies, Santa Clara, CA, United States) equipped with a COSMOSIL C18 column (ID 5 μm ×250 mm) and COSMOSIL Security C18 guard column (ID 5 μm × 250 mm). The flow rate was 1.0 mL/min, and the mobile phase consisted of 55% (v/v) methanol (mobile phase A) and 45% (v/v) water (mobile phase B).

### Antibacterial Activity of Isolated Fungi

The antibacterial activities of all the 48 fungal metabolites were performed. Ten pieces (5 mm in diameter) of the fresh mycelia were inoculated into a 500 mL Erlenmeyer flask containing 150 mL of ME medium and cultured for 7 d at 180 rpm and 28°C. The culture broth filtrate (300 mL) was extracted with ethyl acetate (3 × 300 mL). Evaporation of the menstruum *in vacuo* yielded a solid or oily crude extract that was used for bioassays. The crude extract was dissolved in acetone at a concentration of 6 mg/mL, and 5 μL of the solution was pipetted onto a sterile filter disc (6 mm in diameter), which was placed onto pre-prepared culture medium and sprayed with a suspension of the test organism on the appropriate medium for the respective test organism [28]. The organisms tested were *Staphylococcus aureus* (ATCC 6538), *Bacillus subtilis* (ATCC 6633) and *Escherichia coli* (ATCC 8739). Gentamicin was used as a positive control. Each inhibition assay was repeated three times and estimated by measuring inhibition zone (the citation:-: no activity (<10 mm); +: activity (12–15 mm); ++: good activity (15–20 mm); +++: very good activity (>20 mm).

## Results

### Phylogenetic Diversity of Cultivable Fungi Associated with the Gut of Healthy *P*. *flavescens* Larvae

In total, 48 isolates were obtained from the guts of healthy *P*. *flavescens* larvae. The ITS rDNA region of all fungi were sequenced and the identification was performed by comparison with published sequences in GenBank. Sequence analysis revealed that most of the fungal isolates (46) were grouped into 4 classes (Sordariomycetes, Eurotiomycetes, Leotiomycetes and Dothideomycetes) within the phylum Ascomycota.

The largest number of the fungal isolates belonged to the Dothideomycetes (S1 Fig, S1 Table). Eighteen isolates of the class Dothideomycetes were distributed in the genera *Phoma*, *Curvularia*, *Paraphaeosphaeria*, and *Cladosporium*. In this study, five isolates were highly similar to *Phoma* sp. with an identity of more than 99%. The *Curvularia* included three isolates, and two of the isolates had 100% similarity with *C*. *crepinii*, and another was closely related to *Curvularia* sp. with well-supported sequence alignment. However, there were 8 isolates that had highly similar sequences to an uncultured ascomycete identified from a southern Great Plains mixed grass prairie ecosystem with a sequence similarity of more than 97% with respect to available GenBank reference sequences [29]. A subsequent morphological analysis revealed that the conidia were ovoid or rounded, nonseptate, and pale yellowish brown, which conformed to the description of the *Paraphaeosphaeria* sp. [30]. Thus, we classified these fungi as *Paraphaeosphaeria* sp. The last two isolates belonged to the genus *Cladosporium*, and they were identified as *C*. *cladosporioides*.

Another representative group was Hypocreales, which belong to the class Sordariomycetes. Two species belonged to the genera *Fusarium* and had highly similar sequences to *F*. *chlamydosporum* and *F*. *oxysporum*. The most frequent genus identified in this study was *Trichoderma*. In total, thirteen strains were in the genus *Trichoderma* and were identified as *T*. *asperellum* (1), *Trichoderma* sp. (4), *T*. *citrinoviride* (1), *T*. *gamsii* (2) and *T*. *longibrachiatum* (4). Among them, QTYC-44 exhibited a sequence match of 95% to *T*. *citrinoviride* and did not share the same group according to the phylogenetic analysis, which indicated that this strain may not have been previously sequenced and deposited in the GenBank database or might represent a novel taxon. Finally, one strain exhibited a highly similar sequence to *Hypocrea lixii*.

Fungal isolates belonging to class Eurotiomycetes were assigned to 3 genera of the Eurotiales: *Penicillium* (7), *Aspergillus* (1) and *Neosartorya* (1). The *Penicillium* isolates were identified as *P*. *citrinum* (2), *P*. *oxalicum* (1), *P*. *georgiense* (1) and *Penicillium* sp. (2). Two isolates belonging to the family Aspergillaceae were similar to *A*. *terreus*, with a sequence match of >99%. Finally, one isolate was identified as *N*. *aureolaone*, which had been previously analyzed by Peterson [31].

Leotiomycetes isolates found in this study included two strains that exhibited highly similar sequences (>98%) to *Chaetomella raphigera*. Finally, two strains were grouped in Mucorales (Zygomycete). Both belonged to the genus *Rhizopus* with a high sequence match to *R*. *microsporus* (>99%).

### Diversity of Bacterial Symbionts

In this study, 14 bacterial symbionts were initially observed from 14 fungal genomic DNA extractions based on positive PCR results using 16S primers. Our survey data provided direct evidence that the ability of gut fungi to harbor bacterial symbionts is diverse and phylogenetically widespread. The 14 bacterial 16S DNA sequences were assessed by a BLAST comparison in GenBank. These bacterial symbionts were spread broadly across 3 classes of Ascomycota fungi in our surveys. Among those fungal hosts, four belonged to Sordariomycetes, five belonged to the Eurotiomycetes and five belonged to the Dothideomycetes. All bacterial symbionts were divided into 8 genera by initial BLAST comparisons in GenBank and were putatively identified as *Sphingomonas*, *Methylobacterium*, *Burkholderia*, *Pantoea*, *Enterobacter*, *Leclercia*, and *Serratia*, *Oceanobacillus* (S2 Table), which were included in the Proteobacteria (Alphaproteobacteria, Betaproteobacteria, and Gammaproteobacteria) and Firmicutes. *Enterobacter* was the dominant group of bacterial symbionts associated with the fungi. To confirm the presence of live endobacteria with the living mycelia, the fungal preparations of QTYC-45, were stained with a mixture of dyes (SYTO-9 and propidium iodide). As shown in S2 Fig, mycelia of QTYC-45 harbored a high number of endobacteria that fluoresced green, indicating that the endobacteria were alive.

To our knowledge, this is the first report of *Leclercia* sp., *O*. *oncorhynchi* and *M*. *extorquens* as bacterial symbionts in fungi, which suggests that the bacterial symbionts relationship in the insect gut may be more intimate or of greater complexity than elsewhere. In addition to being plant-fungus fungal endophytes [14, 32], we proved that *Sphingomonas*, *Burkholderia*, *Pantoea*, *Enterobacter* and *Serratia* can also inhabit fungi isolated from the gut of insect. For one isolate (QTYC-45; *Paraphaeosphaeria* sp.), we have successfully isolated the bacterial partner (QTYC-45b; *Pantoea agglomerans*) from the fungus.

### Metabolites Differences in Fungus and Sterile Fungus

The next step was to investigate the difference in metabolites between QTYC-45 and sterile QTYC-45. QTYC-45 was cultivated in the presence of penicillin (100 μg/mL) and total absence of bacteria was proved by light microscopic analyses (S2 Fig). The difference of metabolites between the QTYC-45 and sterile QTYC-45 were distinct. The ethyl acetate extract of QTYC-45 and sterile QTYC-45 exhibited a difference in retention time of 10.8 min (S3 Fig, peak marked with*), indicating that the compound (10.8 min, peak marked with *) was produced by the endofungal bacteria itself. The major peak (S4 Fig, peak marked with *) with a retention time of 7.4 min indicated that the endofungal bacteria have an inhibitory effect on fungal metabolites. A methanol extract of mycelium exhibited a difference in retention time of 7.4 min (marked with *) between QTYC-45 and sterile QTYC-45, indicating that the bacterial symbionts also had an inhibitory effect on intracellular fungal metabolites.

### Antibacterial Activities of the Fungi

Of the 48 assayed fungal extracts, 20 (42%) exhibited antibacterial activity against at least one of the bacterial strains, including *E*. *coli* (ATCC 8739), *B*. *subtilis* (ATCC 6633), and *S*. *aureus* (ATCC 6538) (S3 Table). In general, the fungal extracts were most active against Gram-positive bacteria, as opposed to Gram-negative bacteria. Fourteen isolates (belonging to genera *Trichoderma*, *Neosartorya*, *Hypocrea*, *Fusarium*, and *Paraphaeosphaeria*) exhibited good antibacterial activity against *S*. *aureus*. Fifteen isolates (belonging to genera *Trichoderma*, *Neosartorya*, *Hypocrea*, *Paraphaeosphaeria*, *Curvularia* and *Penicillium*) exhibited good antibacterial activity against *B*. *subtilis*. Interestingly, several fungal isolates of the same ITS type exhibited different antibacterial activity. However, all 48 fungal extracts had weak or no activity against *E*. *coli*.

## Discussion

In conclusion, the diversity of the fungi associated with the *P*. *flavescens* larvae were investigated by culture-dependent methods together accompanied by the analysis of the fungal internally transcribed spacer sequences. 48 fungal isolates were obtained, including 5 classes and 12 different genera. Among these isolates, 4% belonged to the Leotiomycetes, 23% belonged to the Dothideomycetes, 20% belonged to the Eurotiomycetes, 29% belonged to the Sordiaromycetes and 4% belonged to the Zygomycete. In addition, we provided evidence that the fungal harboring of bacterial symbionts is phylogenetically widespread in the gut of *P*. *flavescens* larvae. Fourteen 16S rDNA sequences were obtained. Among these, three bacterial symbionts (*Leclercia* sp., *O*. *soncorhynchi* and *M*. *extorquens*) were reported to reside in the fungi for the first time. Finally, a relatively high proportion of the isolates exhibited good or moderate antibacterial activity, demonstrating that gut-associated fungi have high potential to be a rich source of pharmaceutical leads. The results reported here also expand our knowledge of insect gut fungi.

Discovery of the diversity and ecological roles of insect-associated fungi will be important for understanding insect ecology and evolution. The elucidation of the diversity and ecological importance of bacterial symbionts is also important. The isolates appear to be common but previously overlooked inhabitants of insect-associated fungi. Evidence from McFrederick [33] indicated that bacterial richness decreased with antifungal treatments in the *Megachile rotundata* larval gut. Interestingly, using antibacterial treatments, they also found that bacteria have no effect on fungal communities. McFrederick‘s and our results argue that antifungal treatments should be used to investigate whether the symbiotic relationship between fungi and bacterial symbionts play a role in decreasing bacterial richness. Moreover, our work can serve as the starting point for further studies on bacterial diversity, in which the relationships of bacterial symbionts should be considered.

Bioactive natural products have played a prominent role in the investigation and development of antibiotic drugs and agricultural agents. It is straightforward to screen for antibiotic activity from microorganisms, and such screens have been ongoing for approximately 80 years since Alexander Fleming’s work. However, discovery programs employing natural products have been shown to have a high rediscovery rate [34]. Several novel strategies have been suggested to resolve this problem. Seeking novel microbial habitats that have not been previously well explored is one approach that is deemed to be more likely to produce new functional compounds from microbes [7, 35, 36]. Because the insect gut is an important and novel microbial habitat, many new species have been isolated and identified [21], which provided another important approach for the discovery of new compounds. However, evidence from previous studies indicates that bacterial symbionts can affect the metabolic profile of their host fungi [37]. Their presence leads to the modulation of fungal protein expression, which has an important impact on host metabolism [15, 38]. Exploring the phylogenetic diversity of gut-associated fungi and related bacterial symbionts could be an effective method to discover unknown bioactive molecules from the fungi in the gut of *P*. *flavescens* larvae.

## Supporting Information

S1 Fig Neighbor-joining phylogenetic tree of ITS rDNA sequences of fungal isolates. Bootstrap values were calculated using 1,000 replications. For the closest match related species, the proposed taxonomic names are followed by their respective accession numbers in brackets. Branch lengths are indicated as 0.05 substitutions per positions according to the scale bar underneath the tree.
(TIF)

S2 Fig Laser microscopic investigation of fungal mycelium (A, C: isolate QTYC45; B, D: symbiont-free QTYC45). Mycelium was stained with SYTO 9 from the Live/Dead BacLight Bacterial Viability Kit (Molecular Probes—L7007) and observed using a Leica TCS SP5 AOBS Laser Microscope (Leica Microsystems Ltd., Wetzlar, Germany) at 480/500 nm (A, B: white light and fluorescence mode; C, D: fluorescence mode only). Green fluorescence in the fungal hyphae indicates that bacteria are alive.
(TIF)

S3 Fig HPLC analysis of an ethyl acetate extract of sterile QTYC45 (A treated with penicillin) and QTYC45 (B). The difference in metabolites between fungi and sterile fungi peak were marked with *. mAU, milli absorbance units.
(TIF)

S4 Fig HPLC analysis of a methanol extract of mycelium of sterile QTYC45 (A treated with penicillin) and QTYC45 (B). The difference in metabolites between fungus and sterile fungus peak were marked with *. mAU, milli absorbance units.
(TIF)

S1 Table Phylogenetic affiliations of cultivable fungi associated with *Pantala flavescens* larvae. (DOC)

S2 Table Taxonomic classification for 14 endofungal bacteria. (DOC)

S3 Table Antibacterial activity of 48 fungal metabolite against 3 pathogenic bacteria. (DOC)
