# Supplementary figures and images for: Diversity, Bacterial Symbionts and Antibacterial Potential of Gut-Associated Fungi Isolated from the Pantala flavescens Larvae in China
Source: PLoS One. 2015 Jul 29;10(7):e0134542. doi: 10.1371/journal.pone.0134542 (PMC4519156; doi:10.1371/journal.pone.0134542)

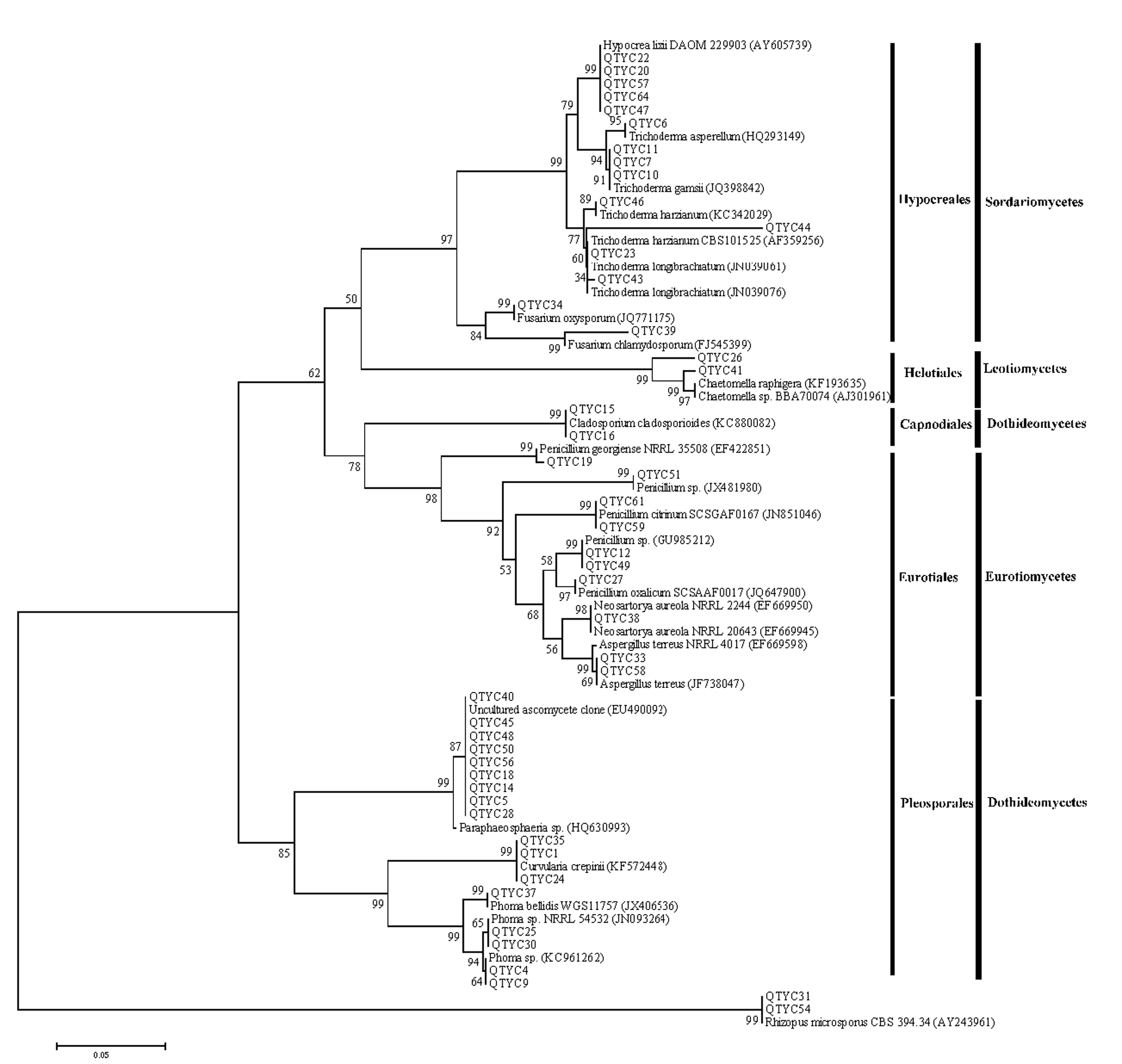

Supplement: S1 Fig — Bootstrap values were calculated using 1,000 replications. For the closest match related species, the proposed taxonomic names are followed by their respective accession numbers in brackets. Branch lengths are indicated as 0.05 substitutions per positions according to the scale bar underneath the tree. (TIF) [file pone.0134542.s001.tif]

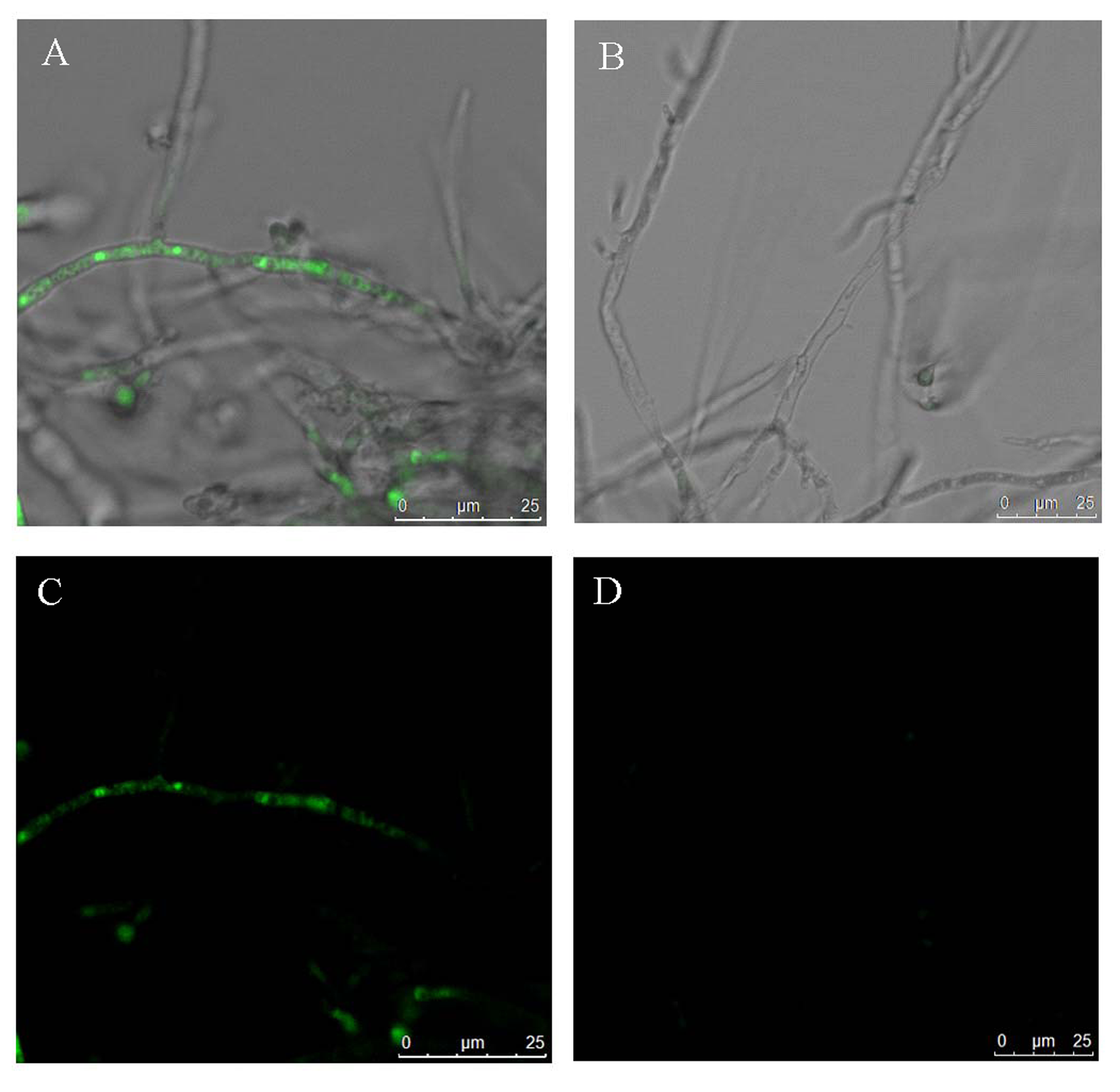

Supplement: S2 Fig — Mycelium was stained with SYTO 9 from the Live/Dead BacLight Bacterial Viability Kit (Molecular Probes—L7007) and observed using a Leica TCS SP5 AOBS Laser Microscope (Leica Microsystems Ltd., Wetzlar, Germany) at 480/500 nm (A, B: white light and fluorescence mode; C, D: fluorescence mode only). Green fluorescence in the fungal hyphae indicates that bacteria are alive. (TIF) [file pone.0134542.s002.tif]

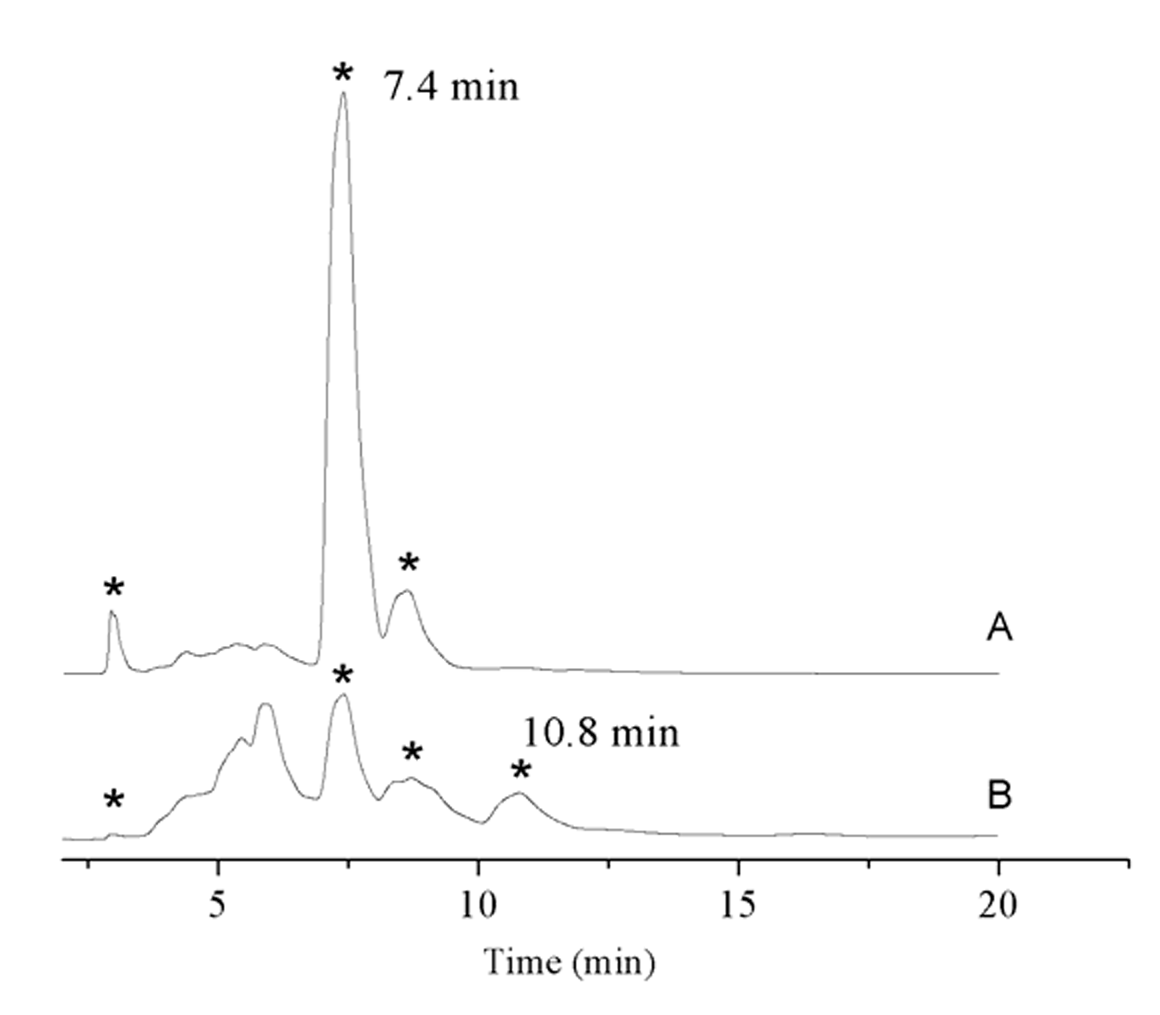

Supplement: S3 Fig — The difference in metabolites between fungi and sterile fungi peak were marked with *. mAU, milli absorbance units. (TIF) [file pone.0134542.s003.tif]

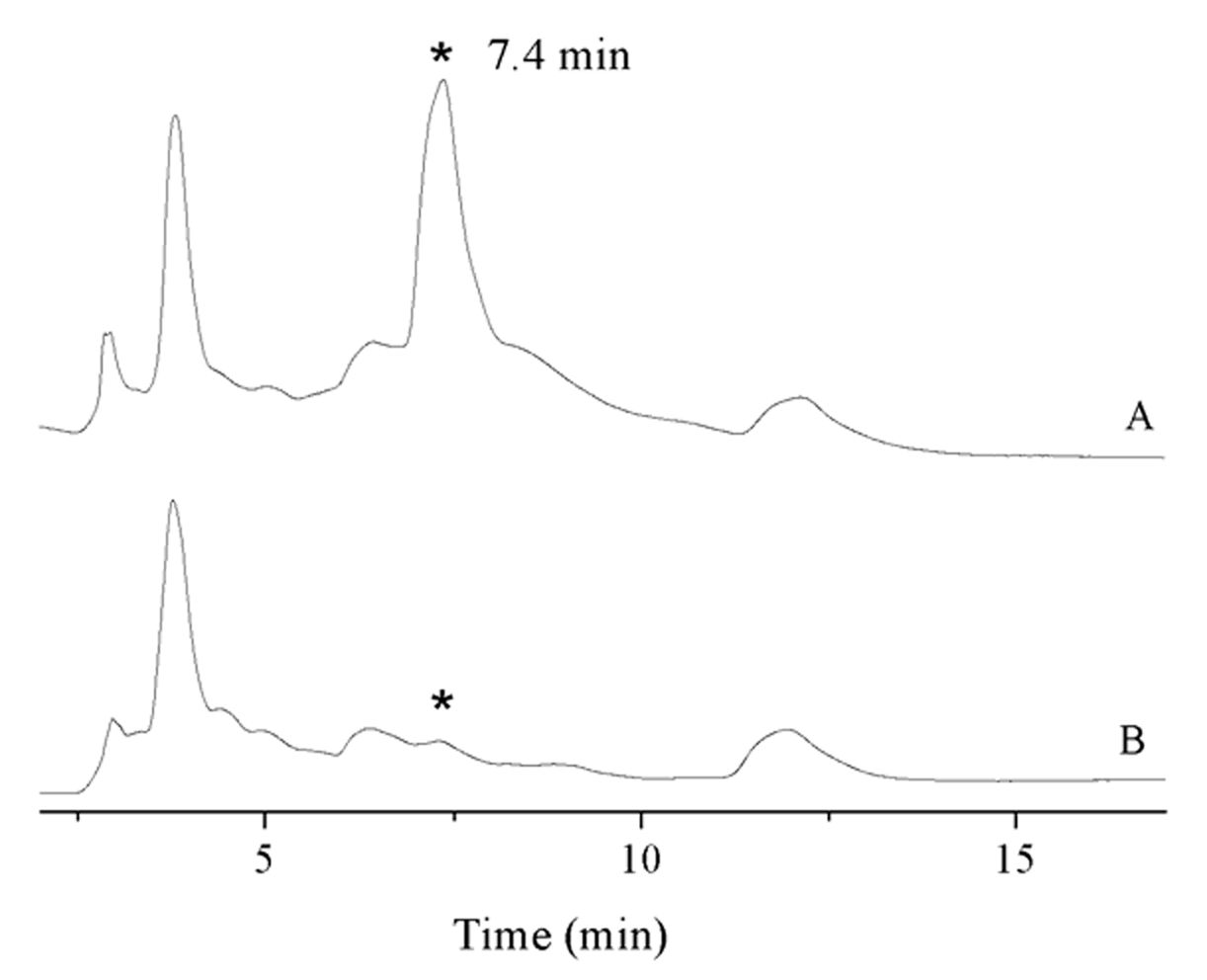

Supplement: S4 Fig — The difference in metabolites between fungus and sterile fungus peak were marked with *. mAU, milli absorbance units. (TIF) [file pone.0134542.s004.tif]
