# Supplementary material for: Diversity, Bacterial Symbionts and Antibacterial Potential of Gut-Associated Fungi Isolated from the Pantala flavescens Larvae in China
Source: PLoS One. 2015 Jul 29;10(7):e0134542. doi: 10.1371/journal.pone.0134542 (PMC4519156; doi:10.1371/journal.pone.0134542)
Supplement: S1 Table — (DOC) [file pone.0134542.s005.doc]

**S1 Table.** **Phylogenetic affiliations of cultivable fungi associated with *Pantala flavescens* larvae**

| **Isolate code** | **Closest match** | **Accession no.** | **Coverage/Max ident** | **GenBank no.** |
| --- | --- | --- | --- | --- |
| **QTYC-33** | *Aspergillus terreus* | JF738047 | 98/99 | KM103344 |
| **QTYC-58** | *Aspergillus terreus* | JF738047 | 98/99 | KM103317 |
| **QTYC-1** | *Curvularia crepinii* | KF572448 | 97/100 | KM103330 |
| **QTYC-35** | *Curvularia crepinii* | KF572448 | 96/100 | KM103329 |
| **QTYC-24** | *Curvularia* sp*.* | HE861835 | 98/99 | KM103325 |
| **QTYC-15** | *Cladosporium cladosporioides* | KC880082 | 99/99 | KM103316 |
| **QTYC-16** | *Cladosporium cladosporioides* | KC880082 | 98/100 | KM103301 |
| **QTYC-26** | *Chaetomella raphigera* | KF193635 | 97/97 | KM103305 |
| **QTYC-41** | *Chaetomella raphigera* | KF193635 | 95/99 | KM103302 |
| **QTYC-39** | *Fusarium chlamydosporum* | FJ545399 | 91/99 | KM103331 |
| **QTYC-34** | *Fusarium oxysporum* | KF673560 | 96/99 | KM103328 |
| **QTYC-64** | *Hypocrea lixii* | EF596951 | 97/99 | KM103336 |
| **QTYC-38** | *Neosartorya aureola* | EF669945 | 99/99 | KM103297 |
| **QTYC-48** | *Paraphaeosphaeria* sp. | JN198486 | 89/99 | KM103304 |
| **QTYC-59** | *Penicillium citrinum* | JN851046 | 97/99 | KM103309 |
| **QTYC-27** | *Penicillium oxalicum* | JQ647900 | 98/99 | KM103315 |
| **QTYC-19** | *Penicillium georgiense* | EF422851 | 98/97 | KM103307 |
| **QTYC-12** | *Penicillium* sp. | GU985202 | 98/99 | KM103340 |
| **QTYC-49** | *Penicillium* sp. | GU985212 | 98/99 | KM103311 |
| **QTYC-51** | *[Paraconiothyrium](http://blast.ncbi.nlm.nih.gov/Blast.cgi#alnHdr_339283353)* sp. | GU055987 | 99/99 | KM103319 |
| **QTYC-61** | *Penicillium citrinum* | JN851046 | 97/99 | KM103312 |
| **QTYC-4** | *Phoma* sp. | KC961262 | 98/99 | KM103310 |
| **QTYC-9** | *Phoma* sp. | KC961261 | 97/99 | KM103332 |
| **QTYC-25** | *Phoma* sp. | HQ696085 | 98/99 | KM103321 |
| **QTYC-30** | *Phoma* sp. | KF177690 | 99/99 | KM103324 |
| **QTYC-37** | *Phoma* sp. | KC928322 | 99/99 | KM103323 |
| **QTYC-31** | *Rhizopus microsporus* | HQ404248 | 99/99 | KM103334 |
| **QTYC-54** | *Rhizopus microsporus* | AY243961 | 98/99 | KM103343 |
| **QTYC-6** | *Trichoderma asperellum* | HQ293149 | 99/99 | KM103338 |
| **QTYC-44** | *Trichoderma citrinoviride* | HM776434 | 76/95 | KM103326 |
| **QTYC-57** | *Trichoderma citrinoviride* | HQ596983 | 96/99 | KM103306 |
| **QTYC-7** | *Trichoderma gamsii* | GQ121324 | 97/100 | KM103339 |
| **QTYC-11** | *Trichoderma gamsii* | JQ398842 | 97/99 | KM103313 |
| **QTYC-10** | *Trichoderma longibrachiatum* | HQ833356 | 99/99 | KM103342 |
| **QTYC-23** | *Trichoderma longibrachiatum* | JN039061 | 99/99 | KM103341 |
| **QTYC-43** | *Trichoderma longibrachiatum* | JN039076 | 98/99 | KM103327 |
| **QTYC-46** | *Trichoderma longibrachiatum* | KC254099 | 97/99 | KM103308 |
| **QTYC-20** | *Trichoderma* sp. | KF367552 | 98/99 | KM103314 |
| **QTYC-47** | *Trichoderma* sp. | JF304977 | 99/99 | KM103335 |
| **QTYC-22** | *Trichoderma* sp. | JF304985 | 98/99 | KM103337 |
| **QTYC-5** | *Paraphaeosphaeria* sp. | EU490092 | 97/97 | KM103320 |
| **QTYC-14** | *Paraphaeosphaeria* sp. | EU490092 | 97/98 | KM103299 |
| **QTYC-18** | *Paraphaeosphaeria* sp. | EU490092 | 98/97 | KM103322 |
| **QTYC-28** | *Paraphaeosphaeria* sp. | EU490092 | 96/98 | KM103300 |
| **QTYC-40** | *Paraphaeosphaeria* sp. | EU490092 | 97/97 | KM103318 |
| **QTYC-50** | *Paraphaeosphaeria* sp. | EU490092 | 98/96 | KM103303 |
| **QTYC-56** | *Paraphaeosphaeria* sp. | EU490092 | 97/98 | KM103298 |
| **QTYC-45** | *Paraphaeosphaeria* sp. | EU490092 | 97/97 | KM103333 |
