# Supplementary material for: Diversity, Bacterial Symbionts and Antibacterial Potential of Gut-Associated Fungi Isolated from the Pantala flavescens Larvae in China
Source: PLoS One. 2015 Jul 29;10(7):e0134542. doi: 10.1371/journal.pone.0134542 (PMC4519156; doi:10.1371/journal.pone.0134542)
Supplement: S2 Table — (DOC) [file pone.0134542.s006.doc]

**S2 Table. Taxonomic classification for 14 endofungal bacteria**

| **Isolate code** | **Closest match** | **Accession no.** | **Coverage/Max ident** | **GenBank no.** |
| --- | --- | --- | --- | --- |
| **QTYC59b** | *Enterobacter aerogenes* | [AB844449](http://www.ncbi.nlm.nih.gov/nucleotide/529367210?report=genbank&log$=nucltop&blast_rank=1&RID=VWCYT1MK015) | 99/99 | KM974654 |
| **QTYC57b** | *Leclercia* sp*.* | [JX174253](http://www.ncbi.nlm.nih.gov/nucleotide/401878605?report=genbank&log$=nucltop&blast_rank=1&RID=VWD7KH06015) | 99/99 | KM974655 |
| **QTYC40b** | *Serratia* sp. | KF501474 | 99/99 | KM974664 |
| **QTYC25b** | *Oceanobacillus oncorhynchi* | DQ089679 | 99/99 | KM974661 |
| **QTYC24b** | *Enterobacter aerogenes* | AB844449 | 99/99 | KM974656 |
| **QTYC46b** | Uncultured bacterium clone AKIW1136 | DQ129349 | 99/99 | KM974663 |
| **QTYC12b** | *Enterobacter aerogenes* | NR-102493 | 99/99 | KM974660 |
| **QTYC56b** | *Burkholderia tropica* | HQ023270 | 99/98 | KM974662 |
| **QTYC51b** | Uncultured *Sphingomonas* sp. | JX575941 | 99/99 | KM974665 |
| **QTYC47b** | *Methylobacterium extorquens* | NR-074138 | 99/99 | KM974666 |
| **QTYC33b** | *Serratia* sp. | KF501474 | 99/99 | KM974657 |
| **QTYC45b** | *Pantoea agglomerans* | KC764985 | 99/99 | KM974667 |
| **QTYC64b** | *Burkholderia unamae* | HQ023246 | 99/99 | KM974658 |
| **QTYC61b** | *Enterobacter aerogenes* | AB844449 | 99/99 | KM974659 |
