# Supplementary material for: Diversity, Bacterial Symbionts and Antibacterial Potential of Gut-Associated Fungi Isolated from the Pantala flavescens Larvae in China
Source: PLoS One. 2015 Jul 29;10(7):e0134542. doi: 10.1371/journal.pone.0134542 (PMC4519156; doi:10.1371/journal.pone.0134542)
Supplement: S3 Table — (DOC) [file pone.0134542.s007.doc]

**Table 3.** **Antibacterial activity of 48 fungal metabolite against 3 pathogenic bacteria.**

| **Isolate No.** | **Proposed identity** | ***S. aureus*** | ***B. subtilis*** | ***E. coli*** |
| --- | --- | --- | --- | --- |
| **QTYC-33** | *Aspergillus terreus* | - | - | - |
| **QTYC-58** | *Aspergillus terreus* | - | - | - |
| **QTYC-1** | *Curvularia crepinii* | - | + | - |
| **QTYC-35** | *Curvularia crepinii* | - | - | - |
| **QTYC-24** | *Curvularia* sp. | - | - | - |
| **QTYC-15** | *Cladosporium cladosporioides* | - | - | - |
| **QTYC-16** | *Cladosporium cladosporioides* | - | - | - |
| **QTYC-26** | *Chaetomella raphigera* | - | - | - |
| **QTYC-41** | *Chaetomella raphigera* | - | - | - |
| **QTYC-39** | *Fusarium chlamydosporum* | ++ | - | - |
| **QTYC-34** | *Fusarium oxysporum* | - | - | - |
| **QTYC-64** | *Hypocrea lixii* | ++ | + | - |
| **QTYC-38** | *Neosartorya aureola* | +++ | + | - |
| **QTYC-48** | *Paraphaeosphaeria* sp. | - | ++ | - |
| **QTYC-59** | *Penicillium citrinum* | - | + | - |
| **QTYC-27** | *Penicillium oxalicum* | - | - | - |
| **QTYC-19** | *Penicillium* sp. | - | + | - |
| **QTYC-12** | *Penicillium* sp. | - | - | - |
| **QTYC-49** | *Penicillium* sp. | - | +++ | - |
| **QTYC-51** | *[Paraconiothyrium](http://blast.ncbi.nlm.nih.gov/Blast.cgi#alnHdr_339283353)* sp. | - | - | - |
| **QTYC-61** | *Penicillium citrinum* | - | - | - |
| **QTYC-4** | *Phoma* sp. | - | - | - |
| **QTYC-9** | *Phoma* sp. | - | - | - |
| **QTYC-25** | *Phoma* sp. | - | - | - |
| **QTYC-30** | *Phoma* sp*.* | - | - | - |
| **QTYC-37** | *Phoma* sp. | - | - | - |
| **QTYC-31** | *Rhizopus microsporus* | - | - | - |
| **QTYC-54** | *Rhizopus microsporus* | - | - | - |
| **QTYC-6** | *Trichoderma asperellum* | + | +++ | - |
| **QTYC-44** | *Trichoderma* sp. | +++ | +++ | - |
| **QTYC-57** | *Trichoderma citrinoviride* | ++ | +++ | - |
| **QTYC-7** | *Trichoderma gamsii* | + | + | - |
| **QTYC-11** | *Trichoderma gamsii* | + | + | - |
| **QTYC-10** | *Trichoderma longibrachiatum* | + | - | - |
| **QTYC-23** | *Trichoderma longibrachiatum* | ++ | ++ | - |
| **QTYC-43** | *Trichoderma longibrachiatum* | + | + | - |
| **QTYC-46** | *Trichoderma longibrachiatum* | - | - | - |
| **QTYC-20** | *Trichoderma* sp. | + | - | - |
| **QTYC-47** | *Trichoderma* sp. | + | - | - |
| **QTYC-22** | *Trichoderma* sp. | - | - | - |
| **QTYC-5** | *Paraphaeosphaeria* sp. | - | - | - |
| **QTYC-14** | *Paraphaeosphaeria* sp. | - | - | - |
| **QTYC-18** | *Paraphaeosphaeria* sp. | - | - | - |
| **QTYC-28** | *Paraphaeosphaeria* sp. | - | - | - |
| **QTYC-40** | *Paraphaeosphaeria* sp. | + | - | - |
| **QTYC-50** | *Paraphaeosphaeria* sp. | - | - | - |
| **QTYC-56** | *Paraphaeosphaeria* sp. | - | - | - |
| **QTYC-45** | *Paraphaeosphaeria* sp. | - | + | - |
| GentamycinSulfate | | +++ | +++ | +++ |
